# E-SegNet: E-Shaped Structure Networks for Accurate 2D and 3D Medical Image Segmentation

**DOI:** 10.34133/research.0869

**Published:** 2025-09-03

**Authors:** Wei Wu, Xin Yang, Chenggui Yao, Ou Liu, Qi Zhao, Jianwei Shuai

**Affiliations:** ^1^ School of Computer Science and Software Engineering, University of Science and Technology Liaoning, Anshan 114051, China.; ^2^ College of Data Science, Jiaxing University, Jiaxing 314000, China.; ^3^ Oujiang Laboratory (Zhejiang Lab for Regenerative Medicine, Vision and Brain Health), Wenzhou Institute, University of Chinese Academy of Sciences, Wenzhou 325001, China.

## Abstract

U-structure has become a foundational approach in medical image segmentation, consistently demonstrating strong performance across various segmentation tasks. Most current models are based on this framework, customizing encoder–decoder components to achieve higher accuracy across various segmentation challenges. However, this often comes at the cost of increased parameter counts, which inevitably limit their practicality in real-world applications. In this study, we provide an E-shaped segmentation framework that discards the traditional step-by-step resolution recovery decoding process, instead directly aggregating multi-scale features extracted by the encoder at each stage for deep cross-level integration. Additionally, we propose an innovative multi-scale large-kernel convolution (MLKConv) module, designed to enhance high-level feature representation by effectively capturing both local and global contextual information. Compared to U-structure, the proposed E-structured approach substantially reduces parameters while delivering superior performance, especially in complex segmentation tasks. Based on this structure, we develop 2 segmentation networks specifically for 2-dimensional (2D) and 3D medical images. 2D E-SegNet is evaluated on four 2D segmentation benchmark datasets (Synapse multi-organ, ACDC, Kvasir-Seg, and BUSI), while 3D E-SegNet is assessed on four 3D segmentation benchmark datasets (Synapse, ACDC, NIH Pancreas, and Lung). Experimental results demonstrate that our approach outperforms the current leading U-shaped models across multiple datasets, achieving new state-of-the-art (SOTA) performance with fewer parameters. In summary, our research introduces a novel approach to medical image segmentation, offering potential improvements and contributing to ongoing advancements in the field. Our code is publicly available on https://github.com/zhaoqi106/E-SegNet.

## Introduction

Image segmentation plays a crucial role in medical image processing and analysis, effectively assisting healthcare professionals in diagnosis. It significantly reduces the learning curve and time investment for medical personnel, providing faster and more precise diagnostic tools that accelerate workflows and enhance the efficiency and accuracy of clinical work. Traditional medical image segmentation relies on mathematical methods such as edge detection, thresholding, and machine learning [1], but these often fall short for complex medical images with diverse types and blurred boundaries. In recent years, deep learning approaches have gained popularity and have been widely applied in various fields of bioinformatics. These applications include prediction of miRNA–lncRNA interactions [2–4], computational toxicology [5–7], metabolite–disease association prediction [8,9], remote health monitoring [10–13], proteomics identification [14], and histopathological image analysis [15–17]. Because of the rapid advancements in deep learning, its performance in medical image segmentation has now far surpassed traditional methods. However, current segmentation algorithms often rely on stacking additional modules to enhance accuracy, leading to model expansion that is challenging to deploy in resource-constrained environments. Consequently, developing a versatile, lightweight, and precise segmentation algorithm has been a driving force in our research.

Since the advent of convolutional neural networks (CNNs) in 2010, they have dominated the field of computer vision. The convolution-based U-Net [18], with its encoder–decoder structure and skip connection technique, has demonstrated considerable advantages in semantic segmentation tasks. Influenced by U-Net, models such as U-Net++ [19], ResUnet [20], Attention U-Net [21], CE-Net [22], Unet3+ [23], and Kiu-Net [24] adopted similar U-structures, leading to significant progress in various medical segmentation tasks and further validating this architecture’s effectiveness. With the rise of Vision Transformer (ViT) [25] in 2020, it has gained widespread recognition in medical image segmentation due to its larger receptive field and powerful modeling capabilities. Following this trend, SwinUnet [26] and AgileFormer [27] leveraged various ViT adaptations, using an inverted encoder structure to construct decoders and forming purely ViT-based U-shaped segmentation networks. Models like UCTransNet [28], EMCAD [29], 2-dimensional (2D) D-LKA [30], and MIST [31] combine ViT encoders with CNN decoders, effectively enhancing both global and local feature modeling. These studies have promoted the rapid development of U-structure framework in 2D medical image segmentation. In 3D medical image segmentation, U-shaped structure is equally widely adopted. Models such as UNETR [32], UNETR++ [33], Swin UNETR [34], nnFormer [35], and D-LKA Net [30] have all utilized this architecture, achieving remarkable results in their respective segmentation tasks.

**Fig. 1.**
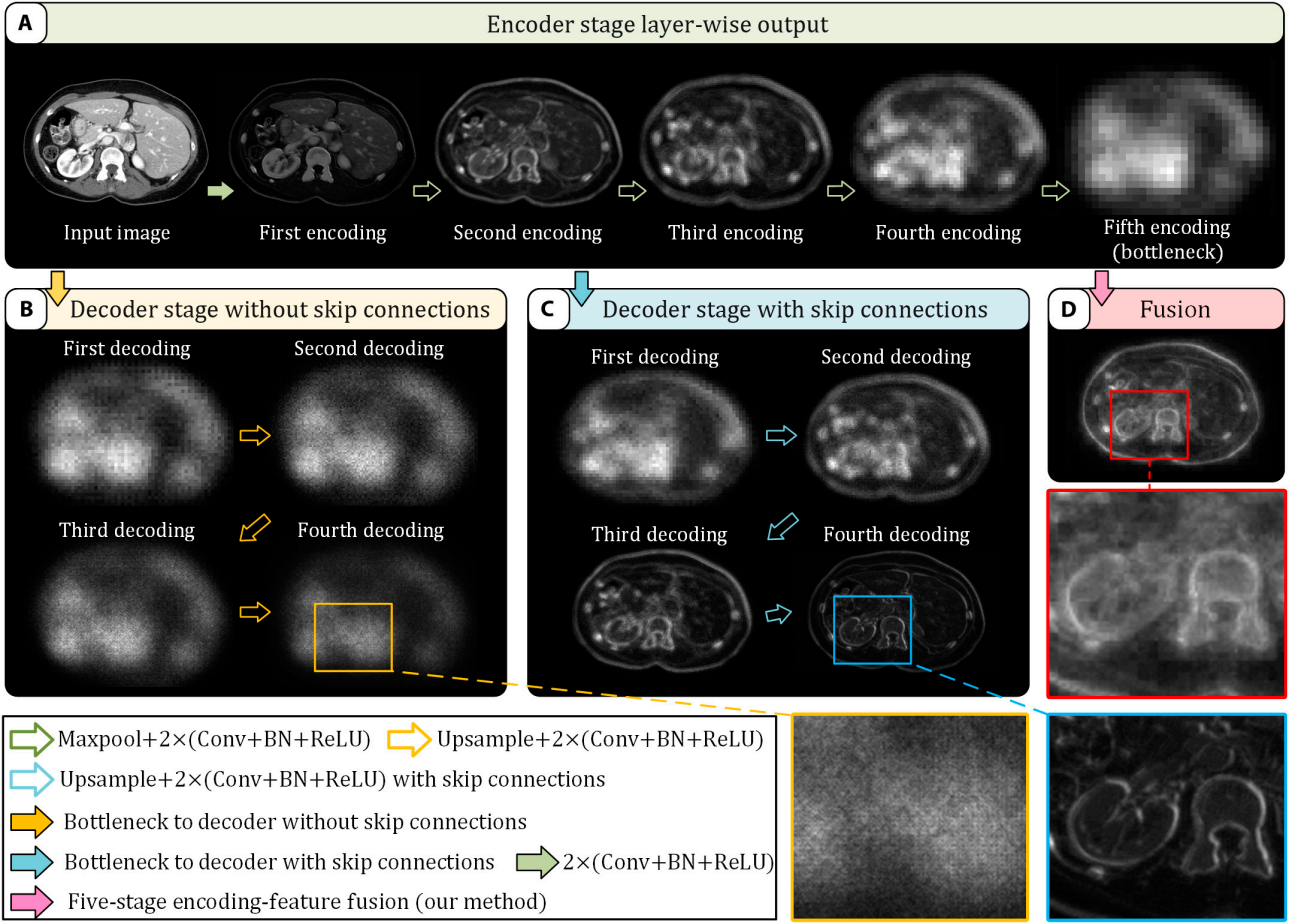
Comparison of feature visualizations between E-structure and traditional U-shaped network [12] at each stage. (A) Visualization of outputs at each layer during the encoding (feature extraction) phase. (B) Visualization of outputs at each layer during the decoding phase of traditional U-shaped network without skip connections. (C) Visualization of outputs at each layer during the decoding phase of traditional U-shaped network with skip connections (U-Net). (D) Visualization of E-structure outputs, where features from all encoding layers are aggregated directly without a decoding phase. Note that (B), (C), and (D) all use the same encoder (A) for feature extraction to enable a qualitative comparison across stages.

However, our research reveals that the symmetric encoder–decoder structure of U-shaped networks inherently demands a large number of parameters, and deeper network layers increase the risk of overfitting during training. For instance, leading models in 2D segmentation tasks on the Synapse dataset now exceed 100 million parameters, nearly 5 times the parameter count of state-of-the-art (SOTA) models from 2020, yet they yield only about a 5% improvement in DSC performance. Analyzing the outputs at each stage of a U-Net on an abdominal computed tomography (CT) image, we observe additional phenomena. Figure 1B presents stage outputs without skip connections, while Fig. 1C shows the standard U-Net output. Without shallow information from skip connections, the decoder’s output becomes overly abstract and lacks detail, prompting further exploration into the role of progressive stage information and skip connections in the decoder. In Fig. 1D, we remove the decoder structure and upsample multi-scale features from each stage of the skip connections to the same resolution, directly aggregating them. Feature map preserves global details more effectively than the traditional U-Net. Comparing the output images in Fig. 1C and D, the former exhibits more pronounced edge contours but loses internal organ details. As semantic segmentation is a pixel-wise classification task, it inherently prioritizes the preservation of fine-grained details, which is more effectively achieved in the latter. However, this focus also leads to the introduction of additional noise and irrelevant pixels. Therefore, a new refinement module is needed after aggregation to extract critical features and eliminate redundant information.

Based on these findings, we propose an E-shaped segmentation structure without conventional progressive decoder. This structure upsamples and aggregates multi-scale features from each encoder stage to the original resolution, and then applies an MLKConv module for fine feature extraction, improving interpixel and interchannel associations. This design enhances segmentation accuracy and feature representation, effectively capturing intricate details in complex scenes. Specifically, our key contributions are as follows:

1. We introduce an E-shaped segmentation architecture that removes conventional step-by-step decoding process, differing from the U-structure and significantly reducing model parameters. A novel MLKConv module is designed, utilizing depthwise convolutions with varying scales and dilation rates to efficiently extract and fuse local and global information with minimal parameters.
2. Based on this E-structure, we construct an efficient 2D segmentation model and evaluate it on 4 different types of public medical datasets. Our model surpasses most current U-structure methods in segmentation accuracy and achieves the lower parameter count and faster inference speed among models with comparable performance.
3. Furthermore, we develop a 3D segmentation model based on this E-structure with ViT as the encoder, demonstrating the structure’s generality across 2D and 3D segmentation tasks. Testing on 4 public 3D medical segmentation datasets, we achieve leading results with fewer parameter count.
4. We re-design several U-shaped networks, adapting them to E-structure, and perform comparative analyses with their original U-structure counterparts. This evaluation validates the extensibility of E-structure by assessing its advantages and limitations.

## Results

To comprehensively evaluate our method, we benchmark 2D E-SegNet on Synapse [36], ACDC [37], Kvasir-SEG [38], and BUSI [39] datasets, and 3D E-SegNet on Synapse, NIH Pancreas [40], ACDC, and the Medical Segmentation Decathlon-Lung [41] datasets against current leading methods. Additionally, we extend some methods to E-structure and conduct comparisons with their original structures.

### Datasets and evaluation metrics

The Synapse multi-organ dataset contains 30 clinical abdominal CT scans with a total of 3,779 axial images. Each CT volume consists of 85 to 198 slices with a resolution of 512 × 512, accompanied by segmentation masks of 13 organs. Our model is trained on 18 cases and evaluated on the remaining 12 cases. Consistent with previous work, we report segmentation performance on 8 abdominal organs: spleen, right kidney, left kidney, gallbladder, liver, stomach, aorta, and pancreas. The ACDC dataset consists of magnetic resonance imaging (MRI) images from various patients, with annotations for left ventricle (LV), right ventricle (RV), and myocardium (Myo). This dataset is divided into 70 training, 10 validation, and 20 testing samples. The NIH Pancreas-CT dataset comprises 82 contrast-enhanced 3D abdominal CT scans focusing on the pancreas region, with each scan manually annotated by experts to delineate pancreatic contours. Among them, 62 scans are used for training and the rest for testing. The Kvasir-SEG dataset focuses on the segmentation of colorectal polyps in endoscopic images, containing 1,000 polyp images along with their respective segmentation masks. The dataset is split into training, validation, and testing sets in an 8:1:1 ratio. The Lung dataset consists of 63 CT volumes for a 2-class segmentation task, aimed at distinguishing lung cancer from the background. The data are split into a 4:1 ratio for training and validation. The BUSI dataset comprises 780 breast ultrasound images annotated for binary segmentation tasks involving normal and tumor regions. These images are collected from 600 female patients and include samples of varying quality and diverse lesion characteristics. Due to the lack of a standardized dataset split or official partitioning protocol, we adopt a 5-fold cross-validation strategy to ensure a fair and robust evaluation. All datasets, except BUSI, follow the official and previously established partitioning protocols.

For the abovementioned datasets, we follow the evaluation metrics used in prior work, including average Dice similarity coefficient (DSC), mean intersection over union (IOU), Jaccard index, average surface distance (ASD), and 95% Hausdorff distance (HD95). DSC, IOU, and Jaccard index assess the overlap between segmentation results and ground truth annotations, where values closer to 1 indicate greater overlap. ASD and HD95 reflect the closeness between the predicted boundary and the true boundary, with smaller values indicating better accuracy. Their formulas are as follows:

$$\mathrm{DSC}=\frac{2|X\cap Y|}{|X|+|Y|},$$ (1)

$$\mathrm{IOU}=\text{Jaccard}=\frac{|X\cap Y|}{|X\cup Y|},$$ (2)

$$\mathrm{ASD}=\frac{1}{|S_{X}|+|S_{Y}|}\left(\sum_{a\in S_{x}}d\left(a,\;S_{Y}\right)+\sum_{b\in S_{Y}}d\left(b,\;S_{X}\right)\right),$$ (3)

$$\mathrm{HD}95=\max\left\{\;\underset{x\in X\;y\in Y}{\sup\;\inf}d\left(x,\;y\right),\underset{y\in Y\;x\in X}{\sup\;\inf}d(y,x)\;\right\}.$$ (4)

In these formulas, *X* and *Y* represent the sets of predicted and ground truth segmentation pixels, respectively, and *S_X_* ​and *S_Y​_* denote the sets of points on the predicted and ground truth segmentation boundaries. The term d(*a*,*S_Y_*) indicates the shortest distance from a predicted boundary point a to the true boundary *S_Y_*, and d(*b*,*S_X_*) represents the shortest distance from *a* ground truth boundary point *b* to the predicted boundary *S_X_*. The operators sup and inf denote the supremum (maximum) and infimum (minimum), respectively. HD95 calculates Hausdorff distance by excluding the largest 5% of distances, thereby reducing the impact of outliers.

### Training strategies

Our model is implemented in Python 3.8 and PyTorch 2.0.1 and tested on an RTX 3090 GPU. For dataset splitting and evaluation metrics, we adhere strictly to the standards established in prior studies. 2D E-SegNet is trained for 400 epochs with a batch size of 8, a base learning rate of 1 × 10^−4^, and the AdamW optimizer. The loss function combines Dice loss *L_dice_* and cross-entropy *L_ce_*, computed as follows:

$$L_{\text{total}}=0.6\times L_{\text{dice}}+0.4\times L_{\text{ce}}.$$ (5)

For 3D E-SegNet, we adopt the training strategy used by nnFormer [35], with a batch size of 2 and a base learning rate of 1 × 10^−4^, using the AdamW optimizer. 3D E-SegNet is trained for 1,000 epochs using image patches of size 128 × 128 × 64, with 250 patches per epoch. The formula for calculating the loss function is as follows:

$$L_{\text{total}}=L_{\text{dice}}+L_{\text{ce}}.$$ (6)

These configurations are slightly adjusted across different datasets to accommodate varying data characteristics. Appendix S1 provides the complete training configurations and an explanation of the different loss weightings applied in 2D and 3D models.

### 2D model comparative experiments

**Fig. 2.**
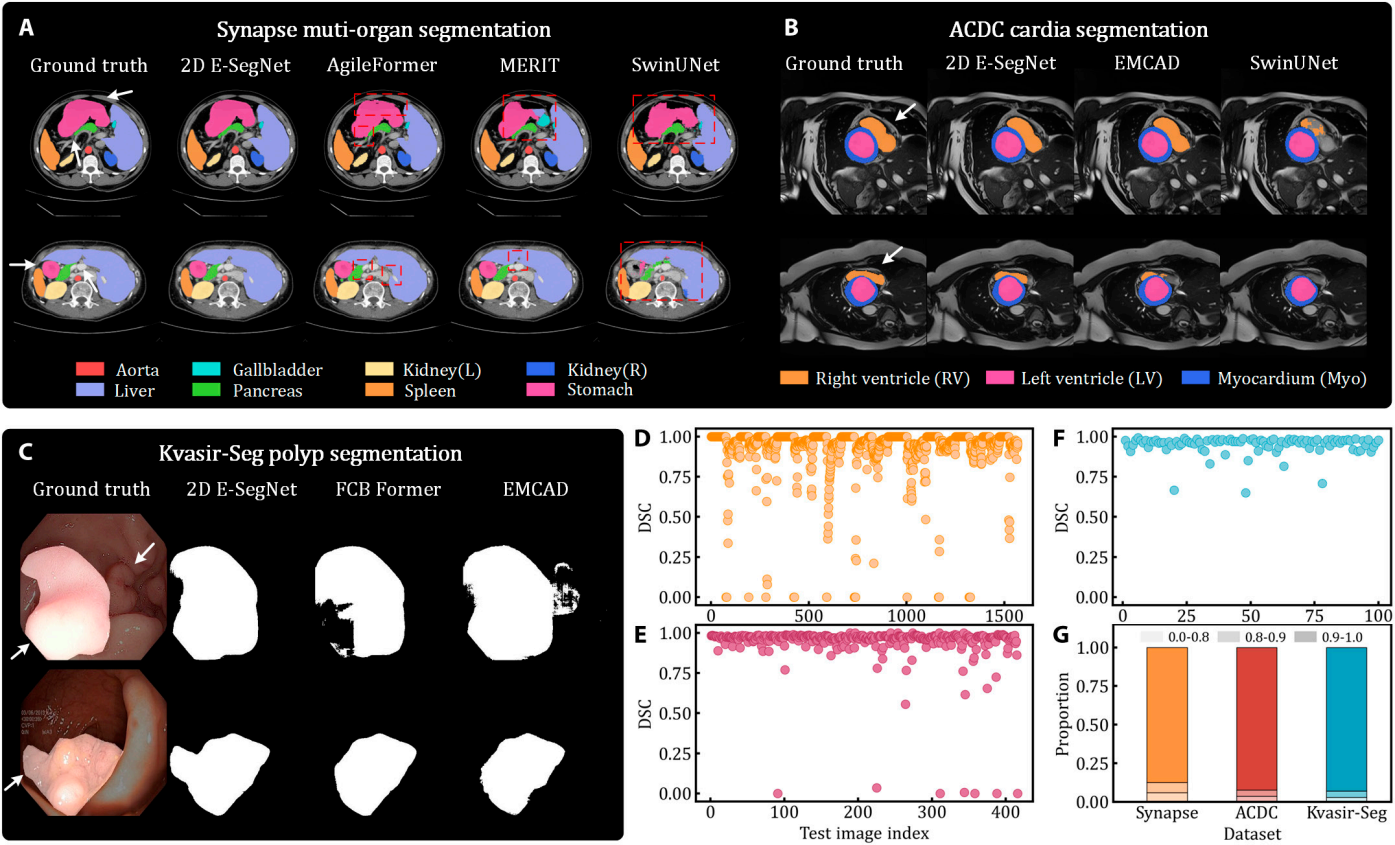
Qualitative and quantitative evaluation of 2D segmentation results. (A) Visualization of multi-organ segmentation on the Synapse dataset, comparing the performance of 2D E-SegNet, AgileFormer, MERIT, and SwinUNet across 8 organs. Different organs are color-coded, and red dashed boxes indicate regions of segmentation failure. White arrows highlight representative areas that are prone to segmentation errors. (B) Visualization of cardiac segmentation on the ACDC dataset, comparing the results of 2D E-SegNet, EMCAD, and SwinUNet for the right ventricle (RV), left ventricle (LV), and myocardium (Myo). (C) Visualization of polyp segmentation results on the Kvasir-Seg dataset, comparing the performance of 2D E-SegNet, FCB Former, and EMCAD. (D to F) Scatterplots of DSC values for test samples on Synapse, ACDC, and Kvasir-Seg datasets, respectively. (G) Proportion of samples within different DSC ranges (0 to 0.8, 0.8 to 0.9, 0.9 to 1) for Synapse, ACDC, and Kvasir-Seg datasets. Due to the presence of samples with missing target region masks in Synapse and ACDC datasets, the background is included in DSC calculation for each sample in (D) and (E).

We conduct a comprehensive comparison of the superior performance achieved by our 2D method against other 2D models on the Synapse dataset. As shown in Table 1, 2D E-SegNet achieves DSC of 86.15% and HD95 of 14.60. This result indicates that 2D E-SegNet demonstrates a clear superiority over previously established leading models, outperforming AgileFormer by 0.41%. Compared to other methods, it exhibits a more substantial advantage. Notably, 2D E-SegNet achieves the best results in segmenting specific anatomical regions, such as the spleen, stomach, aorta, and pancreas. In particular, it improves pancreas segmentation by impressive 2.64% over the second-best method. These regions, characterized by blurred boundaries, irregular shapes, and large spans, have traditionally been challenging for SOTA methods. The significant improvements by our model in these complex target areas indicate its strong advantage in handling intricate segmentations. The qualitative comparison of different methods is shown in Fig. 2A. In the segmentation of the pancreas, liver, and stomach, 2D E-SegNet demonstrates cleaner and more accurate boundaries, significantly reducing misclassifications and better representing the true shape of organs. In contrast, AgileFormer and MERIT exhibit undersegmentation or misclassification in large organs such as the liver and stomach, and fail to accurately delineate small, irregularly shaped organs like the pancreas. SwinUnet performs noticeably worse, producing jagged and fragmented boundaries, particularly in these challenging regions.

**Table 1.** Comparison with 2D models on the Synapse dataset. Bold indicates the best result, and underline represents the second-best result. DSC and HD95 values are averaged across all organs, with individual DSC scores provided for abdominal organs: spleen (Spl), right kidney (Rkid), left kidney (Lkid), gallbladder (Gal), liver (Liv), stomach (Sto), aorta (Aor), and pancreas (Pan).

Methods	DSC (%)↑	HD95↓	Spl	Rkid	Lkid	Gal	Liv	Sto	Aor	Pan
2D E-SegNet	**86.15**	14.60	**92.32**	84.89	87.85	74.51	96.04	**86.75**	**91.37**	**75.48**
AgileFormer [27]	85.74	**7.81**	92.20	85.00	**88.83**	**77.89**	95.64	85.63	89.11	71.62
MERIT [51]	84.90	13.22	92.01	84.85	87.79	74.40	95.26	85.38	87.71	71.81
2D D-LKA Net [30]	84.27	20.04	91.22	84.92	88.38	73.79	94.88	84.94	88.34	67.71
AI-SAM [64]	84.21	12.11	90.32	**85.01**	86.56	74.53	**96.30**	79.24	88.89	72.84
EMCAD [29]	83.63	15.68	92.17	84.10	88.08	68.87	95.26	83.92	88.14	68.51
MISSFormer [46]	81.96	18.20	91.92	82.00	85.21	68.65	94.41	80.81	86.99	65.67
SwinUnet [26]	79.13	21.55	90.66	79.61	83.28	66.53	94.29	76.60	85.47	56.58
MaskDINO [65]	77.64	22.12	88.38	70.40	79.60	68.02	94.20	73.17	87.77	59.61
TransUnet [44]	77.49	31.69	85.07	77.02	81.87	63.16	94.08	75.62	87.23	55.86
U-Net [18]	76.85	39.70	86.67	68.60	77.77	69.72	93.43	75.58	89.07	53.98

The segmentation performance on the ACDC dataset is shown in Table 2. 2D E-SegNet achieves DSC of 92.53%, outperforming the second-best method by 0.21%, with the best performance on RV and Myo segmentations. A qualitative comparison, illustrated in Fig. 2B, shows that 2D E-SegNet more accurately tracks complex contours. For RV region, other methods tend to over- or undersegment, whereas our method provides a more precise segmentation.

**Table 2.** Comparative analysis of 2D E-SegNet and other leading methods on the ACDC dataset, showing the average DSC for segmentation and DSC values for the right ventricle (RV), myocardium (Myo), and left ventricle (LV). Bold indicates the best result.

Methods	Avg.DSC (%)↑	RV	Myo	LV
2D E-SegNet	**92.53**	**91.26**	**90.29**	96.03
MERIT [51]	92.32	90.87	90.00	**96.08**
MERIT-GCASCADE [52]	92.23	90.64	89.96	96.08
EMCAD [29]	92.12	90.65	89.68	96.02
AI-SAM [64]	92.06	90.18	89.94	96.05
PVT-GCASCADE [52]	91.95	90.31	89.63	95.91
TransCASCADE [53]	91.63	89.14	90.25	95.50
MaskDINO [65]	90.08	87.28	87.79	95.17
SwinUnet [26]	90.00	88.55	85.62	95.83
TransUNet [44]	89.71	88.86	84.53	95.73
MISSFormer [46]	87.90	86.36	85.75	91.59

The comparison with leading methods on the Kvasir-SEG dataset is shown in Table 3. 2D E-SegNet achieves DSC of 94.83% and mean IOU of 90.66%, outperforming the second-best method by 0.38% and 0.92%, respectively. The segmentation visualization results are shown in Fig. 2C. 2D E-SegNet produces more complete and accurate segmentation shapes, with sharper boundaries that better preserve the true morphology of polyps, even when lesion regions closely resemble surrounding tissues. In contrast, FCB Former and EMCAD display fragmented and noisy predictions near boundaries. FCB Former tends to undersegment the lesion area, while EMCAD suffers from both under- and oversegmentation, with predictions occasionally spilling into adjacent tissue.

**Table 3.** Comparative analysis of 2D E-SegNet and other leading methods on the Kvasir-SEG dataset, showing the average DSC and average IOU. Bold indicates the best result. The evaluation metrics that are missing in the original paper are denoted by a horizontal line.

Methods	DSC (%)↑	mIOU (%)↑
2D E-SegNet	**94.83**	**90.66**
FCB Former [54]	94.45	89.74
SEP [66]	94.11	90.02
SSFormer-L [67]	93.57	89.05
EMCAD [29]	92.8	-
PVT-GCASCADE [52]	92.74	87.90
PVT-CASCADE [53]	92.58	87.76
TransFuse-L [68]	91.80	86.80
U-Net++ [69]	82.10	-
U-Net [18]	81.80	-

The DSC distribution for test samples on Synapse, ACDC, and Kvasir-Seg datasets is shown in Fig. 2D to F, respectively, while the proportions of DSC values across different intervals are illustrated in Fig. 2G. A significant majority of samples achieve a DSC above 0.9, with 87% on Synapse, 92% on ACDC, and 93% on Kvasir-Seg. Notably, for the Kvasir-Seg dataset, which focuses on polyp pathology segmentation, all results are above 65%. This highlights the robustness and stability of 2D E-SegNet in producing accurate and reliable segmentation results across diverse datasets. These findings demonstrate the strong generalization ability of 2D E-SegNet, ensuring consistent and precise segmentation performance across various 2D tasks and datasets.

The comparison with other methods on the BUSI dataset is shown in Table 4. 2D E-SegNet achieves an average DSC of 81.91 ± 1.47% and a mean IOU of 74.45 ± 1.56%, surpassing the second-best method, AgileFormer, which obtains 78.63 ± 2.56% in average DSC and 70.47 ± 2.70% in mean IOU. Moreover, E-SegNet demonstrates better stability across the 5-fold cross-validation, as evidenced by its lower standard deviation in both metrics.

**Table 4.** Comparative analysis of 2D E-SegNet and other methods on the BUSI dataset, showing the average DSC and average IOU across 5-fold cross-validation. Results are reported as mean ± standard deviation. Bold indicates the best result.

Methods	DSC (%)↑	mIOU (%)↑
2D E-SegNet	**81.91 ± 1.47**	**74.45 ± 1.56**
AgileFormer [27]	78.63 ± 2.56	70.47 ± 2.70
2D D-LKA Net [30]	77.62 ± 1.45	70.35 ± 1.87
EMCAD [29]	76.63 ± 1.18	69.25 ± 1.43
FPN [70]	75.58 ± 2.15	68.32 ± 2.34
MISSFormer [46]	72.36 ± 1.73	65.32 ± 1.59
SwinUnet [26]	71.23 ± 1.12	64.50 ± 1.56
TransUNet [44]	70.58 ± 0.95	64.96 ± 0.99
U-Net++ [69]	68.73 ± 1.66	63.46 ± 1.89
U-Net [18]	69.75 ± 1.71	64.37 ± 1.92

### 3D model comparative experiments

**Fig. 3.**
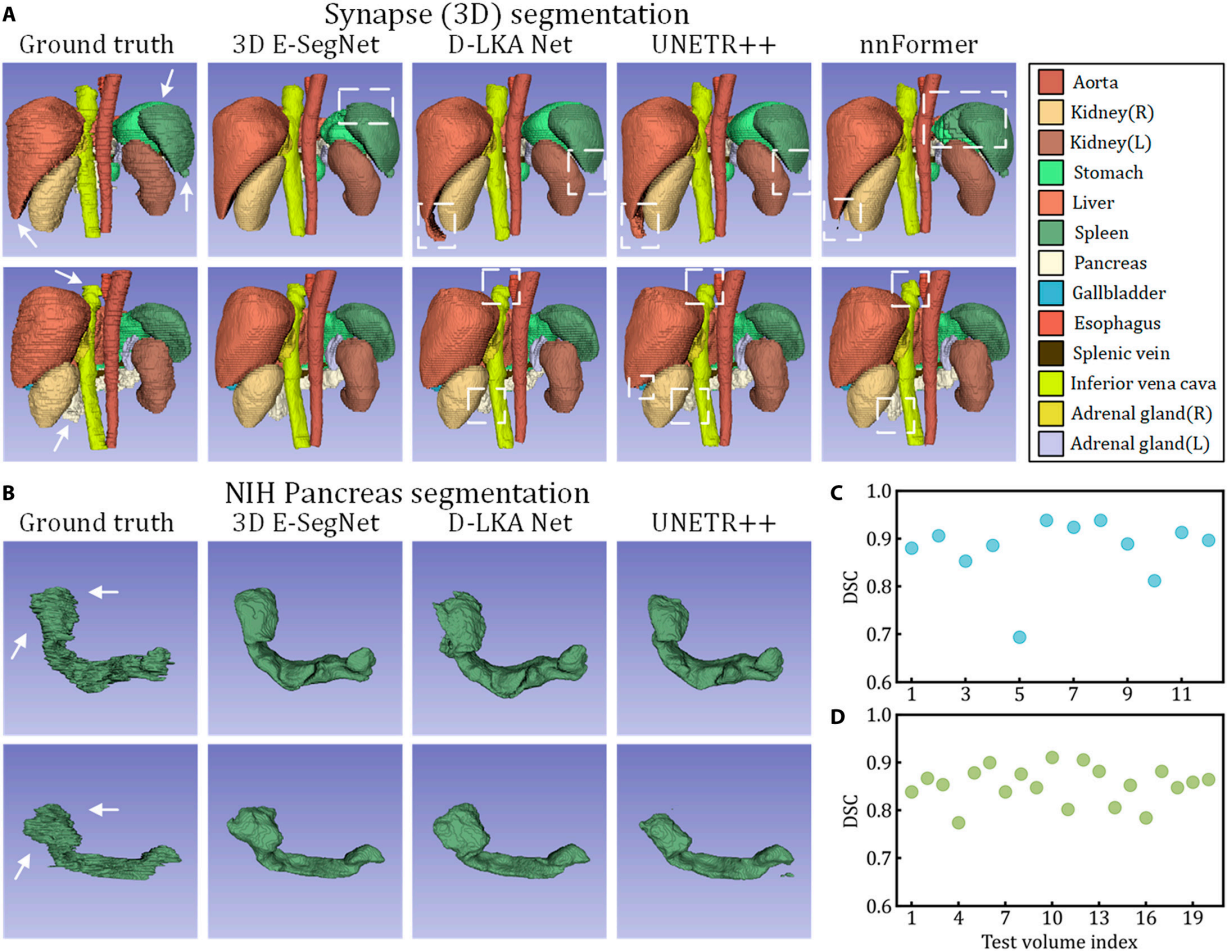
Qualitative and quantitative evaluation of 3D segmentation results. (A) Visualization of multi-organ segmentation on the Synapse (3D) dataset, comparing the performance of 3D E-SegNet, D-LKA Net, UNETR++, and nnFormer across 13 organs. Different organs are color-coded, and white dashed boxes indicate regions of segmentation failure. White arrows highlight representative areas that are prone to segmentation errors. (B) Visualization of pancreas segmentation on the NIH Pancreas dataset, comparing the performance of 3D E-SegNet, D-LKA Net, and UNETR++. (C and D) Scatterplots of DSC values for test samples on the Synapse and NIH Pancreas datasets, respectively.

We compare our 3D method with previous SOTA methods on the Synapse dataset. As shown in Table 5, 3D E-SegNet achieves DSC of 87.76% and HD95 of 6.32, ranking first in both metrics. Compared to D-LKA Net (the previous leading method), it achieves a DSC improvement of 0.27% and an HD95 improvement of 3.25. Performance is slightly improved on left kidney and significantly enhanced on gallbladder and liver. The 3D segmentation visualizations on the Synapse dataset are shown in Fig. 3A. 3D E-SegNet demonstrates greater spatial consistency and volume fidelity, particularly in the segmentation of liver, pancreas, and inferior vena cava. In contrast, D-LKA Net and UNETR++ tend to produce volumetric inflation of liver, with visible overextension into surrounding regions. Meanwhile, nnFormer and UNETR++ underrepresent pancreas and inferior vena cava, leading to noticeable discontinuities in the reconstructed structures. While 3D E-SegNet, like other methods, also exhibits mild oversegmentation in the spleen, it better preserves the overall anatomical structure and continuity across adjacent organs.

**Table 5.** Comparison with 3D models on the Synapse dataset. Bold indicates the best result, and underline represents the second-best result.

Methods	DSC (%)↑	HD95↓	Spl	Rkid	Lkid	Gal	Liv	Sto	Aor	Pan
3D E-SegNet	**87.76**	**6.32**	95.50	86.91	**87.66**	**73.94**	**97.26**	86.43	92.73	81.66
D-LKA Net [30]	87.49	9.57	**95.88**	**88.50**	87.64	72.14	96.25	85.03	92.87	81.64
UNETR++ [33]	87.22	7.53	95.77	87.18	87.54	71.25	96.42	86.01	92.52	81.10
nnU-Net [63]	86.99	10.78	91.86	88.18	85.57	71.77	97.23	85.26	**93.01**	83.01
nnFormer [35]	86.57	10.63	90.51	86.25	86.57	70.17	96.84	**86.83**	92.04	**83.35**
Swin UNETR [34]	83.48	10.55	95.37	86.26	86.99	66.54	95.72	77.01	91.12	68.80
UNETR [32]	78.35	18.59	85.00	84.52	85.60	56.30	94.57	70.46	89.80	60.47

The comparison of segmentation results on the NIH Pancreas dataset is shown in Table 6. 3D E-SegNet outperforms the current SOTA methods across all 4 metrics: DSC, Jaccard, HD95, and ASD. Notably, compared to the previous best results, 3D E-SegNet achieves remarkable improvements of 4%, 5.56%, 3.31, and 0.89 in these metrics, respectively. A qualitative comparison of different methods in Fig. 3B demonstrates that our model has a clear advantage in capturing the overall structure of the pancreas, accurately following the highly irregular shape of the organ.

**Table 6.** Comparative analysis of 3D E-SegNet and other leading methods on the NIH Pancreas dataset, with DSC, Jaccard, HD95, and ASD metrics reported. Bold indicates the best result.

Methods	DSC (%)↑	Jaccard (%)↑	HD95↓	ASD↓
3D E-SegNet	**85.22**	**74.46**	**4.28**	**1.10**
D-LKA Net [30]	81.22	68.90	7.59	1.99
UNETR++ [33]	80.59	68.08	8.63	2.25
UNETR [32]	77.42	63.95	15.07	5.09

The DSC distribution for test samples on Synapse (3D) and NIH Pancreas datasets is shown in Fig. 3C and D, respectively. All samples achieve a DSC above 60%, with most values concentrated around 80% to 90%, closely aligning with the average performance on both datasets. This highlights the strong capability of 3D E-SegNet to deliver accurate segmentation across multiple organs, demonstrating robustness and stability in handling the diverse anatomical and structural complexities inherent to 3D medical imaging.

The comparison of the segmentation results with advanced 3D models on larger datasets is shown in Table 7. On the Lung and ACDC datasets, 3D E-SegNet achieved average DSCs of 81.77% and 92.92%, respectively, outperforming the previous best method, UNETR++, by 1.09% and 0.09%.

**Table 7.** Comparison with leading 3D models on the Lung and ACDC dataset. The horizontal line indicates evaluations that were not recorded.

Methods	Lung	ACDC (3D)			
	DSC (%)↑	Avg.DSC↑	RV	Myo	LV
3D E-SegNet	**81.77**	**92.92**	91.29	90.58	**96.90**
UNETR++ [33]	80.68	92.83	**91.89**	**90.61**	96.00
nnFormer [35]	77.95	92.06	90.94	89.58	95.65
UNETR [32]	73.29	86.61	85.29	86.52	94.02
nnUNet [63]	74.31	91.61	90.24	89.28	95.36
SwinUNETR [34]	75.55	-	-	-	-

### Computational efficiency

**Fig. 4.**
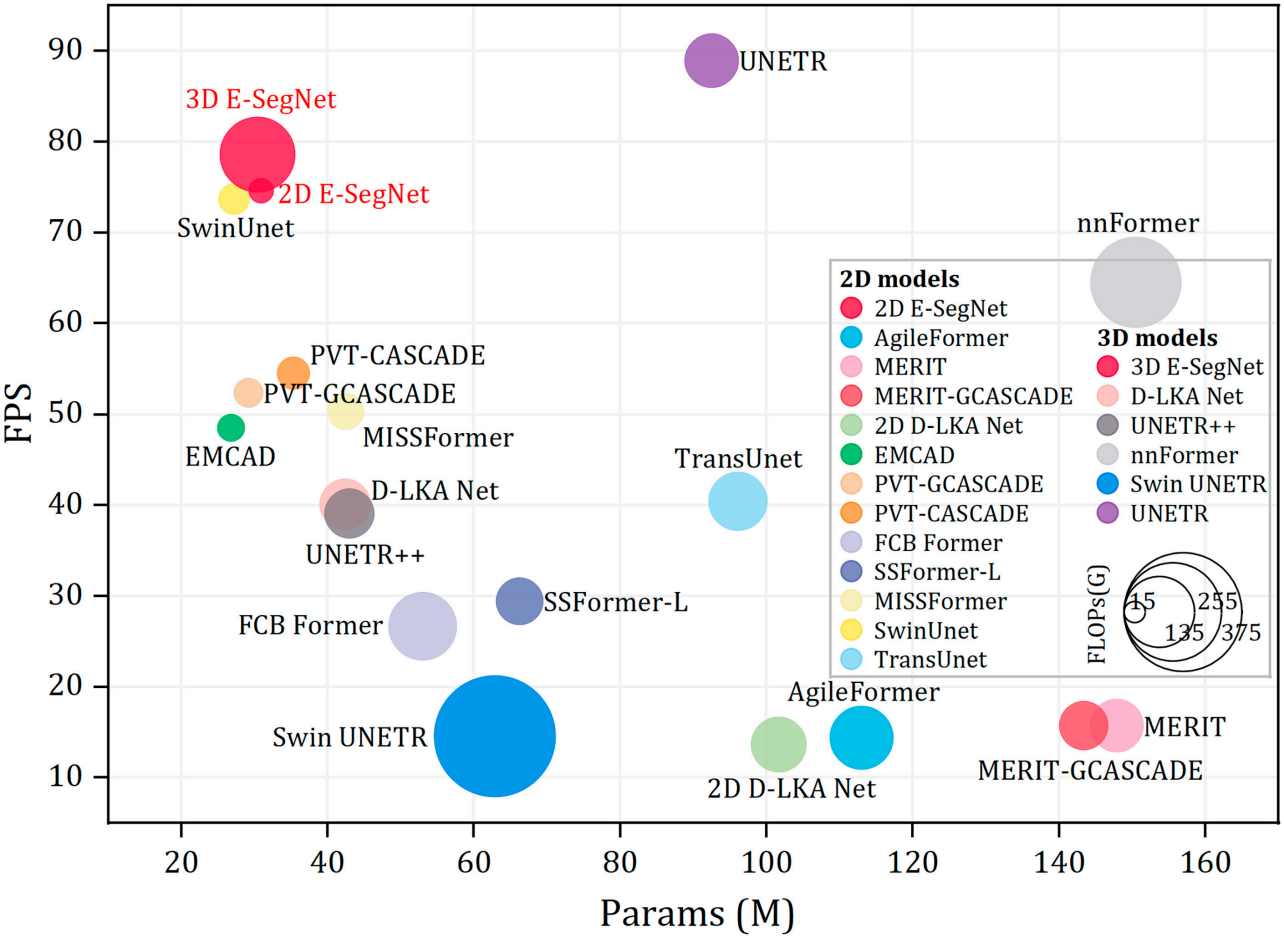
Computational efficiency comparison of 2D (A) and 3D (B) segmentation models in terms of inference speed (FPS), computational complexity (FLOPs), and parameter count (Params). The size of markers represents FLOPs.

We conduct a comprehensive evaluation of computational performance and resource utilization of E-SegNet and other advanced methods. As shown in Fig. 4, in 2D segmentation tasks, 2D E-SegNet achieves a high inference speed of 74.63 frames per second (FPS) with 30.88M parameters and 15.77 GFLOPs (giga floating-point operations per second). Compared to SwinUNet, which has similar computational performance (27.17M parameters, 24.56 GFLOPs, 73.71 FPS), 2D E-SegNet achieves 7.02% and 2.53% higher segmentation accuracy on Synapse and ACDC datasets, respectively. Compared to AgileFormer, which delivers comparable segmentation performance, 2D E-SegNet reduces parameters and FLOPs by 73% and 85%, respectively, and improves inference speed by 418%. In 3D segmentation tasks, 3D E-SegNet maintains good parameter efficiency (31.74M) and inference speed (78.62 FPS), representing improvements of 28.24% and 96.06%, respectively, compared to D-LKA Net (42.35M parameters, 40.10 FPS). Compared with UNETR (92.49M parameters, 88.96 FPS), which offers similar computational performance, E-SegNet achieves 9.41%, 7.8%, 8.48%, and 6.31% higher segmentation accuracy across 4 datasets. Nevertheless, its advantage in FLOPs is less pronounced, mainly due to the high-channel encoder structure and voxel-level operations. Additional details on computational efficiency and resource usage are provided in Appendix S2.

### Ablation study

We further investigate the impact of multi-stage feature aggregation on segmentation performance within the E-structure by conducting additional tests on both 2D and 3D E-SegNet using the Synapse dataset. As shown in Table 8, stage 1 represents the output of stem layer in 2D E-SegNet and the patch embedding layer in 3D E-SegNet, while stages 2 to 5 refer to the outputs of each subsequent MobileNet block or Video Swin Transformer stage, respectively. For example, [1–5] indicates cross-scale aggregation across all stages, including the stem or patch embedding layer, whereas [4,5] represents aggregation of only the last 2 stages. Our evaluation reveals that aggregating all stages achieves the highest DSC in 2D E-SegNet model, while aggregating only stages 3, 4, and 5 results in the lowest HD95. In 3D E-SegNet, the best results for DSC and HD95 are achieved by aggregating all stages except for the patch embedding layer. However, when the patch embedding layer is included in the aggregation, segmentation performance significantly declines. This performance drop can be attributed to the fact that the initial feature representations from the patch embedding layer are relatively coarse and superficial, lacking the rich spatial details and semantic context learned in the later stages of the network. Therefore, including these early-stage features introduces noise into the aggregation, and their alignment with the outputs from the subsequent layers of the Transformer network is typically poor. This misalignment interferes with the finer, more advanced features from the later stages, weakening the model’s ability to capture fine-grained details, particularly in complex anatomical structures, thereby reducing segmentation performance.

**Table 8.** Performance metrics (average DSC and HD95) of 2D E-SegNet and 3D E-SegNet across different stages of polymerization. Numbers in brackets represent the stages of polymerization (e.g., [1–5] includes all feature extraction stages). Bold indicates the best result.

Stage of polymerization	2D E-SegNet		3D E-SegNet	
	DSC (%)↑	HD95↓	DSC (%)↑	HD95↓
[1–5]	**86.15**	14.60	43.03	58.76
[2–5]	85.54	13.01	**87.76**	**6.32**
[1–4]	84.68	15.40	40.88	60.34
[3–5]	85.48	**9.65**	86.88	6.94
[4,5]	85.59	17.38	85.67	8.45

The effect of different refinement strategies on the modeling capacity of aggregated features is also examined through a set of ablation experiments, where the default MLKConv module is replaced with alternative designs. The compared configurations include (a) the proposed MLKConv module; (b) a DoubleConv block consisting of 2 consecutive blocks of convolution (3 × 3), batch normalization (BN), and ReLU; (c) an SE-enhanced convolutional block (SE + BasicConv), consisting of an SE module and a 3 × 3 convolution with BN and ReLU; and (d) removal of the refinement module entirely (None). These replacements are applied only to the refinement path while maintaining a consistent backbone across all models. For 3D tasks, the modules are implemented using their corresponding 3D counterparts.

As shown in Table 9, removing the refinement module leads to a significant decline in model performance. Specifically, in 2D task, DSC drops by 15.8% and HD95 increases by 22.75; in 3D task, DSC drops by 2.44% and HD95 increases by 2.69. These results indicate that further modeling of aggregated features is critical for accurate detail restoration. Compared with conventional convolutional and attention-enhanced modules, MLKConv achieves superior performance in both 2D and 3D tasks, demonstrating its effective contribution to the model.

**Table 9.** Impact of different refinement modules on segmentation performance. Comparison of MLKConv, DoubleConv, SE + BasicConv, and without refinement module (None) in both 2D and 3D E-SegNet models.

Refinement module	2D E-SegNet		3D E-SegNet	
	DSC (%)↑	HD95↓	DSC (%)↑	HD95↓
MLKConv	**86.15**	**14.60**	**87.76**	**6.32**
DoubleConv	84.86	18.16	86.55	7.64
SE + BasicConv	71.91	26.84	86.72	7.04
None	70.35	37.35	85.32	9.01

### Scalability of E-structure

**Fig. 5.**
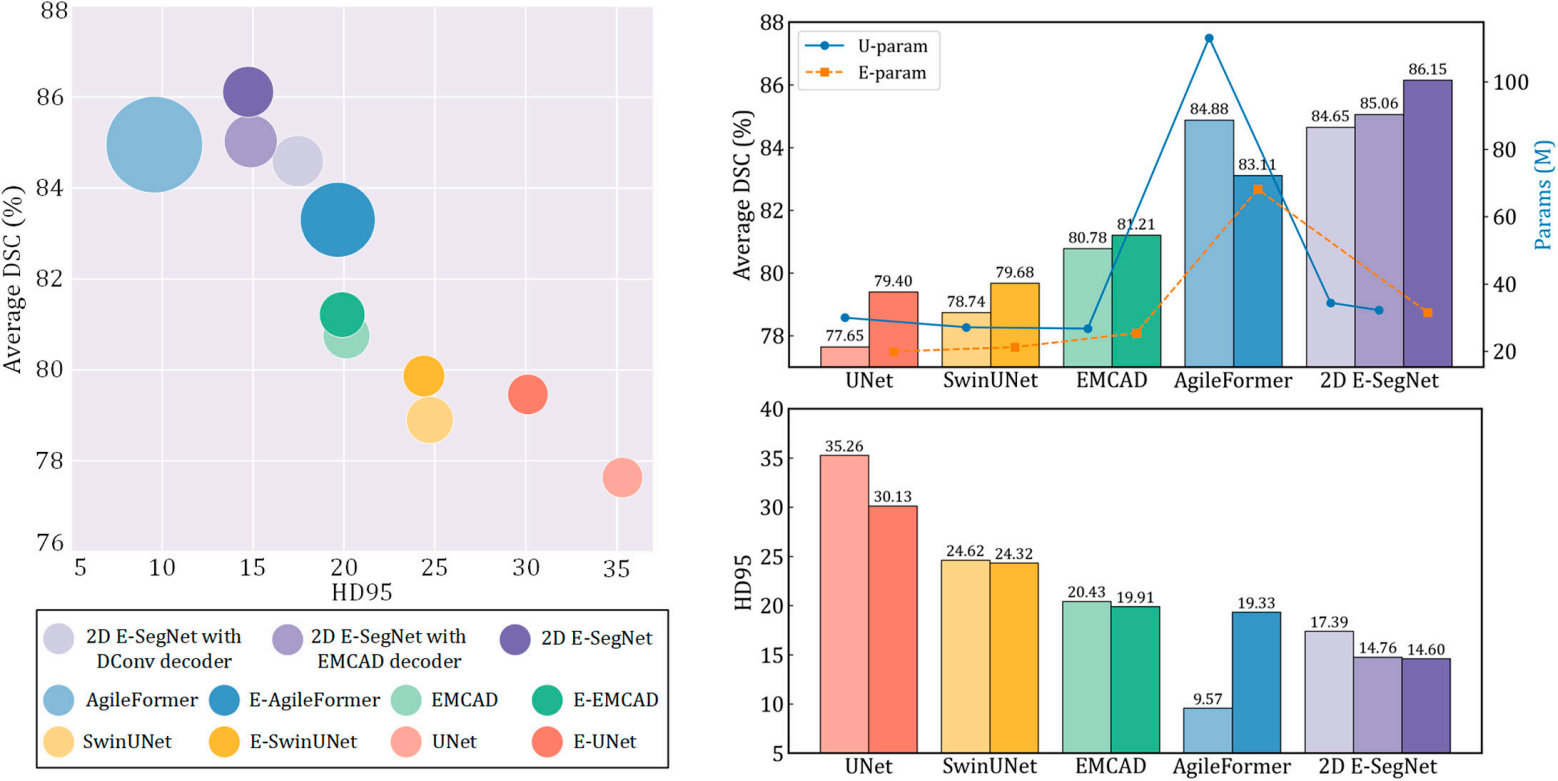
Comparison of E-structure and U-structure on the Synapse dataset, with darker colors indicating E-structure modifications. Larger points in the left figure represent models with greater numbers of parameters.

To validate the applicability of E-structure across different scenarios, we compare the segmentation performance of 5 models using both U-structure and E-structure variants on the Synapse dataset. Specifically, we select the most lightweight and effective EMCAD [29] and DConv decoders (the latter is a modification of UNet decoder and is still widely used) to extend 2D E-SegNet into U-shaped structures. Additionally, we adapt 4 well-known U-structure models (UNet, SwinUnet, EMCAD, and AgileFormer) to E-structure using our approach. As shown in Fig. 5, models with an “E” prefix indicate E-structure adaptation, such as E-UNet, representing UNet extended to E-structure. The results show that the adapted UNet, EMCAD, and SwinUnet have a reduction in parameters of 33%, 5%, and 22%, respectively, compared to their original versions, while their DSC improves by 1.75%, 0.43%, and 0.94%. Although AgileFormer shows a performance drop with a 1.77% decrease in DSC, 40% reduction in parameters makes this trade-off acceptable given the substantial reduction in model size. For 2D E-SegNet, U-structure configurations incorporating EMCAD and DConv decoders result in parameter increases of 4% and 12%, respectively, compared to E-structure configuration, with DSC values decreasing by 1.09% and 1.5%, respectively.

## Discussion

### Model performance and clinical applicability

Deep learning has significantly improved the accuracy of medical image segmentation, yet more efficient, compact, and faster models remain highly advantageous for practical applications. This study proposes a novel architecture, E-SegNet, which establishes new SOTA benchmarks across multiple 2D and 3D medical image segmentation tasks. E-SegNet exhibits strong robustness in challenging scenarios, including blurred boundaries, substantial size variation, and complex anatomical shapes. It achieves lower parameter counts, faster inference speed, and stable performance under most common medical image disturbances (Appendix S3), demonstrating a well-balanced trade-off between performance, efficiency, and model complexity.

The outstanding performance of E-SegNet can be attributed to its innovative E-structured design. Unlike the conventional U-shaped paradigm, E-SegNet removes the progressive resolution recovery process in the decoder, which effectively reduces information loss during cross-layer feature propagation. The introduction of a multi-scale feature aggregation mechanism further enhances feature representation and retains more detailed information. Moreover, the novel MLKConv module, combining multi-scale depthwise separable convolutions and dilated convolutions, addresses the trade-off between parameter size and receptive field in conventional convolutions while optimizing interchannel feature similarity and associations to significantly enhance segmentation accuracy and boundary detail fidelity. Experimental results show that the E-shaped framework adapts well to both 2D and 3D segmentation tasks. Applying the E-structure to conventional U-shaped models further demonstrates its potential as a general design for medical image segmentation and provides valuable insights for future model development.

The performance of E-SegNet suggests broad translational potential in precision medicine. For example, in tumor detection, its accurate delineation of lesions with blurred boundaries can enhance the sensitivity and specificity of early recognition, thereby improving patient prognosis. In pathological biomarker assessment, its fine modeling capability for complex tissue structures helps improve the accuracy of quantitative analysis. In tissue morphology analysis, E-SegNet can assist pathologists in efficient morphological evaluations, significantly reducing the workload of manual annotation. To enable broader clinical applications, our model also needs to adapt to more imaging modalities [such as positron emission tomography (PET) and x-ray] to cope with their differing characteristics (e.g., high noise in PET and low contrast in x-ray). This diversity in imaging characteristics not only demands architectural flexibility but also imposes constraints on computational resources and real-time processing requirements in clinical environments. The inference speed and structural simplicity of E-SegNet provide a solid foundation for further model compression, hardware acceleration, and edge deployment. Translating E-SegNet into clinical practice also necessitates careful attention to ethical and regulatory constraints. Ensuring patient data privacy and compliance with medical data protection standards [such as Health Insurance Portability and Accountability Act (HIPAA) and General Data Protection Regulation (GDPR)] is fundamental, particularly when deploying artificial intelligence (AI) models in cross-institutional or cloud-based settings. Additionally, potential biases introduced by imbalanced training data could impact model generalizability and fairness across patient populations. To foster responsible adoption, interpretability and human oversight are essential, especially in high-stakes diagnostic scenarios. Continuously advancing in these directions will help translate laboratory research into clinical practice, thereby improving diagnostic efficiency and patient care quality.

### Challenges and future perspectives

Despite these advancements, E-SegNet still has certain limitations and areas for improvement. 3D E-SegNet uses a video Swin Transformer encoder, which faces high memory consumption and computational overhead during training. Although it achieves high throughput during inference, FLOPs and memory usage remain relatively large. Compared to its 2D counterpart, it processes voxel-level inputs with much higher dimensionality, resulting in reduced batch size and slower convergence, especially when combined with the self-attention operations in ViT-based encoders. This window-based attention lacks sufficient capacity for long-range spatial modeling. Although E-shaped structure alleviates this limitation through multi-scale feature aggregation, it may still affect global contextual representation in high-resolution scenarios. This could be particularly relevant in segmenting large-area anatomical regions, where maintaining spatial continuity across distant slices is often beneficial for accurate reconstruction. The performance of E-shaped structure heavily relies on the encoder quality and the effectiveness of feature aggregation module. When the encoder design is too simple or the feature fusion is insufficient, segmentation accuracy may decrease in low-contrast or noisy images. Most current 3D segmentation models focus more on the overall architecture rather than optimizing individual encoder–decoder components. As a result, features extracted by the encoder are often not sufficiently refined, leading to little improvement in segmentation performance when applying E-structure to existing U-shaped 3D models. Moreover, the current convolutional upsampling module is relatively basic in terms of feature alignment, and the use of fixed-size kernels in MLKConv module may limit the model’s focus on extremely small or large features.

To further enhance the generality and efficiency of E-SegNet, future research can proceed in multiple directions. Small organ segmentation (such as pancreas and gallbladder) remains a persistent challenge in medical image segmentation due to small voxel proportions. Small targets are highly sensitive to positional information and boundary details, while the current E-SegNet has not introduced dedicated mechanisms for such structures. Future improvements could involve multi-scale context guidance, object-aware loss functions, or saliency learning strategies based on structural priors to enhance the model’s ability to recognize and model small structures. In terms of encoder selection, more lightweight and hardware-friendly architectures can be explored, along with techniques such as model quantization and knowledge distillation to optimize training and deployment efficiency. To further address the limited long-range modeling capacity of encoders, attention mechanisms such as MaxViT or deformable attention could be considered, as they offer enhanced spatial dependency modeling across distant regions. For the upsampling strategy, stronger alignment methods such as attention mechanisms or feature offset strategies could be employed to improve feature fusion accuracy. Regarding MLKConv module, deformable or dynamically sized multi-scale convolution mechanisms could be explored to enable a more adaptive receptive field. In addition, training efficiency could be improved through techniques such as mixed-precision training and gradient checkpointing, which help reduce memory usage and speed up convergence during 3D model optimization. Exploring hybrid forms combining E-SegNet with other architectures also holds significant potential. For example, incorporating graph neural networks to model the topological relationships of anatomical structures could improve the modeling of connected regions such as vessels and nerves. Integrating diffusion models could enhance robustness against high-noise images by generating more reliable feature representations. The Kolmogorov–Arnold networks, through learnable activation functions, can improve nonlinear representation capability, which is suitable for complex tissue modeling. Fast Fourier transform can be used to capture texture information in the frequency domain, improving recognition of periodic structures such as myocardium. The Mamba architecture, with its linear-complexity state-space model, enables long-range dependency modeling while maintaining efficiency, making it suitable for high-resolution medical images.

In addition, the lack of publicly available cross-modal datasets currently restricts our ability to comprehensively evaluate the cross-modal generalization of E-SegNet (e.g., training on MRI and testing on ultrasound). To address this, constructing benchmark multi-modal datasets and developing cross-modal feature alignment mechanisms will be key to enabling reliable knowledge transfer across imaging domains. Other critical aspects of clinical translation include validating model robustness on multi-center datasets with heterogeneous imaging protocols and ensuring system-level compatibility with real-time clinical workflows and deployment hardware.

In conclusion, the structural concept and experimental validation of E-SegNet provide not only a new solution for medical image segmentation tasks but also a foundation for building practical and scalable medical image models. We hope that this study can offer theoretical reference and practical value for future work, further promoting the integration and application of medical AI models in clinical settings.

## Materials and Methods

### Related work

#### 2D medical image segmentation

Over the past decade, with the advent of U-Net [18], CNN-based U-shaped networks have demonstrated significant potential in medical image segmentation. To address feature misalignment issues in skip connections, Attention U-Net [21] and UCTransNet [28] introduced attention mechanisms into the skip connections, while U-Net++ [19] and U-Net3+ [23] employed denser skip connections. Additionally, modifications to the original convolutional module were proposed, such as incorporating residual connections [20,42] and deformable convolutions [43], and KiU-Net [24] added an auxiliary branch to generate richer detail information to supplement U-Net.

Since the introduction of ViT in 2020, it has gained popularity in medical image segmentation due to its unrestricted receptive field and superior ability to capture long-range dependencies between image patches through multi-head self-attention mechanism, surpassing conventional convolutions. TransUNet [44] and TransBTS [45] adopted ViT to replace CNN encoder and bottleneck layer, but the high parameter count and computational overhead of ViT led to significant overfitting issues. With further exploration of ViT variants, models like SwinUnet [26], AgileFormer [27], and MISSFormer [46] have integrated and optimized these ViT modules to construct pure ViT-based U-shaped segmentation models. Additionally, hybrid models combining ViT and CNN (e.g., HiFormer [47,48]) have been progressively refined, while EMCAD [29] and MIST [31] introduced more efficient convolutional decoders paired with ViT encoders, effectively enhancing global information capture and local feature modeling capabilities. Furthermore, cascaded structures have proven particularly effective in refining multi-level features. DS-TransUNet [49] achieved cross-scale feature fusion through parallel Swin Transformer encoders [50] of different scales, enabling better multi-level information capture in medical images. MERIT [51], G-CASCADE [52], and PVT-CASCADE [53] introduced efficient cascaded decoders, achieving higher segmentation accuracy and finer detail restoration for complex images by progressively decoding and layer-wise feature refinement. Additionally, some studies have taken alternative approaches, optimizing loss functions [54] to improve boundary and multi-scale feature capture, and incorporating adaptive pruning strategies [55] to reduce computational costs, thereby better addressing the complexity and resource constraints inherent in medical image segmentation.

#### 3D medical image segmentation

Medical images from MRI or CT scans are typically stored in 3D format. Compared to 2D models, 3D models can more comprehensively capture contextual information and maintain spatial coherence between slices, achieving a cohesive representation of overall structures.

3D U-Net [56], H-DenseUNet [57], and 3D Attention U-Net [58] extended 2D models into 3D space, enabling the capture of spatial information in medical images. Building on this, V-Net [59] introduced residual structures to enhance feature propagation, while LKAU-Net [60] incorporated large convolutional kernels to increase the spatial receptive field. nnFormer [35] designed a pure Transformer-based 3D segmentation architecture, focusing on global modeling of 3D volumetric data. Hybrid architectures like UNETR [32], Swin UNETR [34], and CoTr [61] adopted ViT + CNN combinations, effectively capturing long-range dependencies while preserving local detail. SegFormer3D [62] used a lightweight Transformer structure combined with convolutional layers to avoid the high computational complexity typically associated with traditional Transformers. nnU-Net [63] optimized model structure and training processes through an adaptive configuration mechanism, enhancing compatibility across various medical image segmentation tasks. UNETR++ [33] further improved upon UNETR by incorporating a lightweight Transformer encoder, multi-scale feature fusion, and an enhanced decoder design, achieving more efficient and accurate segmentation. D-LKA Net [30] introduced deformable large-kernel attention mechanisms to strengthen segmentation performance for complex anatomical structures.

Current research predominantly focuses on innovations at module level, often overlooking the impact of overall architecture design. In fact, an optimized model architecture can fundamentally address many persistent issues in medical image segmentation. Our experimental results suggest that the proposed E-structured segmentation network may offer advantages over conventional U-shaped architectures, particularly in capturing targets with diverse sizes and shapes in complex segmentation scenarios.

### Overview of E-SegNet

#### Multi-scale large-kernel convolution

**Fig. 6.**
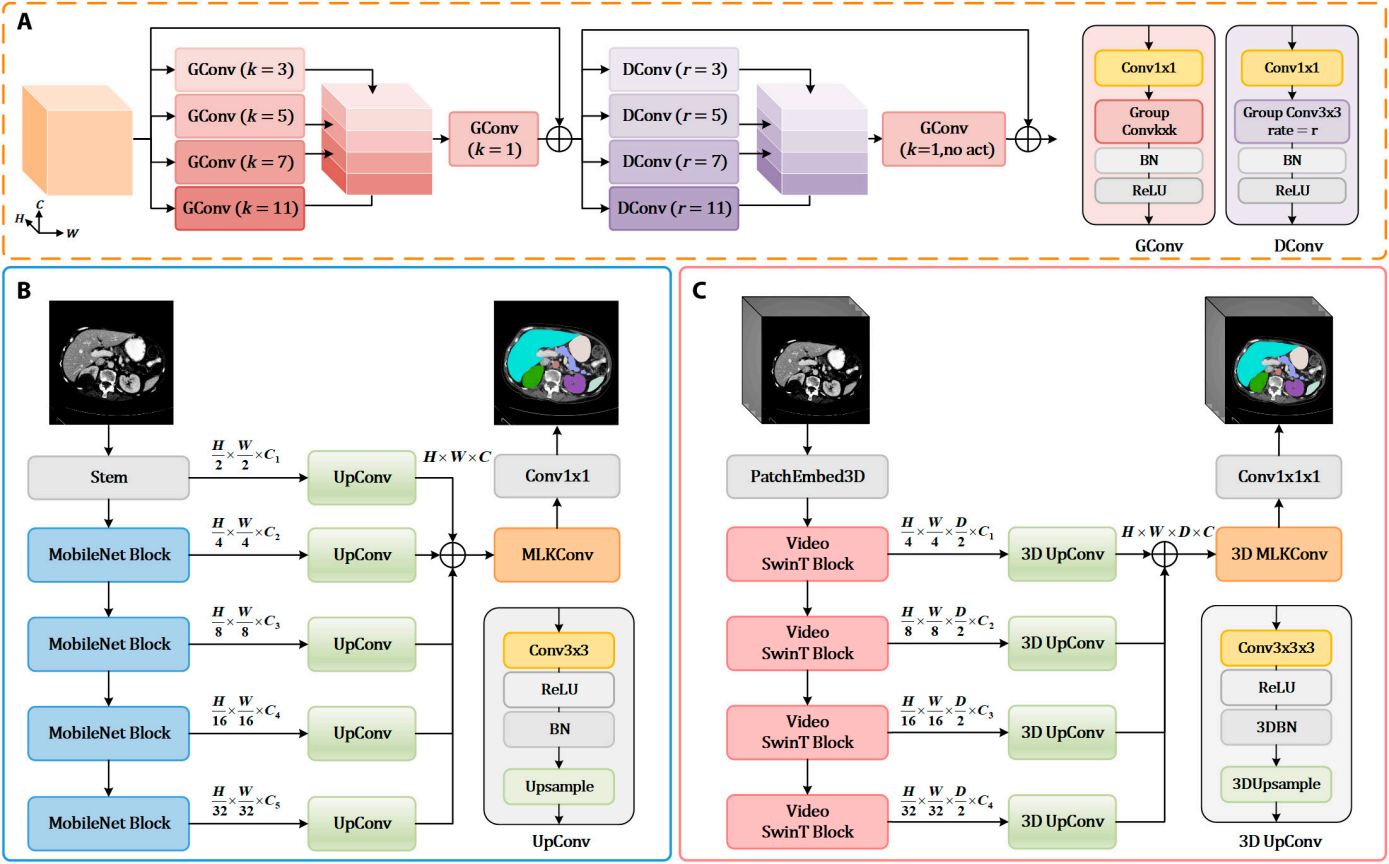
The network architecture of (A) MLKConv, (B) 2D E-SegNet, and (C) 3D E-SegNet.

Multi-scale large-kernel convolution (MLKConv) comprises multi-scale depthwise separable convolutions (GConv) and dilated depthwise separable convolutions (DConv). This design enables efficient extraction and fusion of features across different receptive fields with minimal parameters and computation, enhancing the model’s ability to capture targets of irregular shapes and sizes, which is a crucial aspect in medical imaging. As shown in MLKConv of Fig. 6A, the input features (dimension *H* × *W* × *C*) first pass through 4 GConv modules with kernel sizes of 3, 5, 7, and 11, producing 4 feature maps of dimension *H* × *W* × *C*/4. These maps are then concatenated along the channel dimension to restore the original size, followed by a GConv module with kernel size of 1 to reinforce channel dependencies. Finally, a residual connection ensures effective feature propagation at deeper layers. Each GConv module consists of a 1 × 1 convolution (pointwise convolution, PWConv), a *k* × *k* group convolution (here, using depthwise convolution, DWConv), a BN layer, and a ReLU activation function. This process can be represented as:

$${\text{GConv}}_{k}\left(f\right)=\text{ReLU}\left(\mathrm{BN}\left(\text{DWCon}v_{k}\left(\text{PWConv}\left(f\right)\right)\right)\right),$$ (7)

$$f_{1}=\text{concat}\left({\text{GConv}}_{3}\left(f\right),\;{\text{GConv}}_{5}\left(f\right),\;{\text{GConv}}_{7}\left(f\right),\;{\text{GConv}}_{11}\left(f\right)\right),$$ (8)

$$f_{2}=f+{\text{GConv}}_{1}\left(f_{1}\right),$$ (9)

where GConv*_k_* denotes a GConv module in which the depthwise convolution uses a kernel size of *k*. *f* represents the input feature. Specifically, *f*_1_ and *f*_2_ denote the feature concatenation captured under different receptive fields and the feature channel regularization with residual connections, respectively. In Eq. 2, 1 × 1 convolution in each GConv reduces the number of channels to ^1^/_4_ of the original, while in Eq. 3, 1 × 1 convolution in GConv maintains the number of channels unchanged.

To enhance the model’s long-range modeling capability for large targets in medical images, we introduce group convolutions with varying dilation rates in DConv module to replace standard depthwise convolutions. This approach, processed similarly to GConv, enables the capture of global information over a larger range. Notably, to maintain higher resolution and finer feature representation in the model output, we omit ReLU activation function in the final layer of GConv module. This process can be represented as:

$${\text{DConv}}_{k,r}\left(f_{2}\right)=\text{ReLU}\left(\mathrm{BN}\left({\text{DWConv}}_{k,r}\left(\text{PWConv}\left(f_{2}\right)\right)\right)\right),$$ (10)

$$\begin{array}{c} f_{3}=\\ \text{concat}\left({\text{DConv}}_{3,3}\left(f_{2}\right),\;{\text{DConv}}_{3,5}\left(f_{2}\right),\;{\text{DConv}}_{3,7}\left(f_{2}\right),\;{\text{DConv}}_{3,11}\left(f_{2}\right)\right), \end{array}$$ (11)

$$\text{output}=f_{2}+{\text{GConv}}_{1}\left(f_{3}\right),$$ (12)

where DConv*_k,r_* denotes a DConv module in which the depthwise convolution uses a kernel size of *k* and a dilation rate of *r*. Specifically, *f*_3_ denotes the feature concatenation captured under different, larger receptive fields via dilated convolutions, and output represents the final feature output of MLKConv module. In Eq. 5, 1 × 1 convolution in each DConv reduces the number of channels to ^1^/_4_​ of the original, while in Eq. 6, 1 × 1 convolution in GConv maintains the number of channels unchanged.

The parameter requirements for GConv and DConv can be expressed as follows:

$$P\left(\text{GCon}v_{k}\right)=C_{\text{in}}C_{\text{out}}+\left(k^{2}+2\right)C_{\text{out}},$$ (13)

$$P\left(\text{DCon}v_{3,r}\right)=C_{\text{in}}C_{\text{out}}+11C_{\text{out}},$$ (14)

where *C_in_* and *C_out_* denote the input and output channels, respectively. Thus, the total parameter count for MLKConv is approximately 4*C*^2^ + 70*C*, which is notably lower than a conventional 3 × 3 convolution (with 9*C*^2^ parameters) in practical applications.

#### 2D E-SegNet architecture

The 2D network structure is shown in Fig. 6B. We use MobileNet V4 as the encoder to achieve efficient and precise feature extraction. Formally, the encoder generates 5 multi-scale feature maps at each stage, progressively reducing the image resolution from *H* × *W* to *H*/32 × *W*/32. Each stage’s resolution is half of the previous one, with the shallow stages having higher resolution to capture local structures and edge details, while the deeper stages provide lower resolution but richer channel information, capturing global features such as semantics and overall object shape. These multi-scale features from each stage are adjusted to a uniform size using a convolutional upsampling (UpConv) module. This module first compresses the features with a 3 × 3 convolution, ReLU layer, and BN layer to remove redundant semantic information, preventing mismatch during feature aggregation. Next, nearest-neighbor interpolation restores the features to the original image resolution. The 5 processed features of the same size are then summed element-wise. To further enhance feature representation, we apply MLKConv module to strengthen interpixel relationships, followed by a 1 × 1 convolution to output the final segmentation map. This process can be represented as:

$$U\left(x_{i}\right)=\mathrm{NNI}\left(\mathrm{BN}\left(\text{Relu}\left({\text{Conv}}_{3\times 3}\left(x_{i}\right)\right)\right)\right),$$ (15)

$$X_{\text{out}}={\text{Conv}}_{1\times 1}\left(\mathrm{MLK}\left(U\left(x_{0}\right)+U\left(x_{1}\right)+U\left(x_{2}\right)+U\left(x_{3}\right)+U\left(x_{4}\right)\right)\right),$$ (16)

where Conv_*k*×*k*_ denotes a *k* × *k* convolution operation, NNI denotes nearest-neighbor interpolation, U(*x_i_*) denotes convolutional upsampling operation applied to the output feature from the *i*th stage of encoder, and MLK denotes the MLKConv operation.

#### 3D E-SegNet architecture

As shown in Fig. 6C, 3D E-SegNet uses video Swin Transformer as its encoder. Unlike 2D E-SegNet, 3D E-SegNet divides the input image into multiple 3D patches through a 3D patch embedding layer, mapping these patches into a lower-dimensional feature space, directly reducing the original spatial resolution from *H* × *W* × *D* to *H*/4 × *W*/4 × *D*/2. In the following 4 stages, the first stage keeps the feature shape constant, while the in-plane spatial resolution is progressively halved at each subsequent stage, with the slice-axis dimension remaining unchanged (from *H*/4 × *W*/4 × *D*/2 to *H*/32 × *W*/32 × *D*/2), effectively preserving interslice information, especially in medical images with limited slice counts. The multi-scale spatial features from the encoder’s 4 stages are restored to the original resolution *H* × *W* × *D*, using a 3D convolutional upsampling (3D UpConv) module and aggregated through element-wise addition. This is followed by a 3D MLKConv to further enhance feature representation. Notably, 3D UpConv and 3D MLKConv are 3D extensions of 2D UpConv and 2D MLKConv, adapted for the characteristics of 3D medical imaging. Finally, a 1 × 1 × 1 3D convolution transforms the features into the segmentation map format. This process can be represented as:

$$T\left(x_{i}\right)=\text{TNNI}\left({\mathrm{BN}}_{3D}\left(\text{Relu}\left({\text{Conv}}_{3\times 3\times 3}\left(x_{i}\right)\right)\right)\right),$$ (17)

$$X_{\text{out}}={\text{Conv}}_{1\times 1\times 1}\left({\mathrm{MLK}}_{3D}\left(T\left(x_{1}\right)+T\left(x_{2}\right)+T\left(x_{3}\right)+T\left(x_{4}\right)\right)\right),$$ (18)

where TNNI ​​represents 3D nearest-neighbor interpolation, BN_3D_ denotes a 3D BN layer, T(*x_i_*) indicates 3D convolutional upsampling operation applied to the output feature from the *i*th stage of encoder, and MLK_3D_ denotes the 3D MLKConv operation.

## Nomenclature

**U-shaped models:** A class of encoder–decoder architectures (e.g., U-Net) designed for image segmentation tasks, characterized by symmetric downsampling and upsampling paths with skip connections to recover spatial resolution.
**CNN:** Convolution-based neural networks are widely used in visual recognition and segmentation tasks, capable of learning hierarchical spatial features through stacked convolutional layers and local receptive fields.
**ViT:** A transformer-based architecture that models image patches as sequences, enables effective global context capture without relying on convolutional operations.
**SOTA:** Refers to the best performance or most advanced approach reported in the literature for a given task at the time of evaluation.
**Encoder:** The downsampling path of a segmentation model is used to extract increasingly abstract and semantic features by reducing spatial dimensions and increasing receptive fields.
**Decoder:** The upsampling path of a segmentation model is responsible for progressively recovering spatial resolution and reconstructing the segmentation mask from encoded features.
**Skip connections:** Lateral links between corresponding encoder and decoder stages in U-shaped architectures are designed to fuse low-level spatial features with high-level semantic information.
**Cascade structure:** A parallel multi-branch design within the encoder or decoder, where each branch models features in different ways (e.g., using different resolutions or network architectures), enables richer feature representation in medical image segmentation.
**2D medical image segmentation:** Segmentation tasks perform on individual image slices (e.g., CT or MRI), treating each 2D slice independently.
**3D medical image segmentation:** Volumetric segmentation tasks consider spatial context across multiple slices (e.g., using 3D convolution or attention across depth, height, and width dimensions).
**FPS:** Indicates the speed at which a model processes input images or volumes, commonly used to evaluate inference efficiency in real-time or clinical settings.
**Five-fold cross-validation:** A model validation strategy divides the dataset into 5 subsets, using 4 for training and 1 for testing in rotation, thereby improving the robustness of performance estimates.
**Backbone:** The core feature extractor in a deep learning model, often composes of pretrained architectures (e.g., ResNet and Swin Transformer), which generates multi-level feature representations for downstream tasks like segmentation.
**Stem (stem layer):** The initial convolutional block of a neural network is responsible for basic spatial feature extraction from input images, typically before entering deeper stages of the encoder.
**Video Swin Transformer:** A hierarchical transformer-based backbone is originally designed for video action recognition, adapted here to capture long-range dependencies and spatiotemporal context in 3D medical volumes via shifted window self-attention.
**Stages of aggregation:** Refers to the module where multi-scale features (from all encoder levels) are spatially aligned (e.g., by upsampling) and fused to produce enhanced representations for segmentation. In E-SegNet, this replaces traditional decoder stages.
**MaxVit:** Combines convolutional operations with both block-wise and grid-wise attention to effectively capture spatial dependencies across scales, offering a unified and efficient architecture for vision tasks.

## Data Availability

The codes and datasets are available online at https://github.com/zhaoqi106/E-SegNet.

## Supplementary Materials

Supplementary 1 Appendices S1 to S3 Figs. S1 and S2
