# Supplementary material for: E-SegNet: E-Shaped Structure Networks for Accurate 2D and 3D Medical Image Segmentation
Source: Research (Wash D C). 2025 Sep 3;8:0869. doi: 10.34133/research.0869 (PMC12408157; doi:10.34133/research.0869)
Supplement: Supplementary 1 — Appendices S1 to S3 Figs. S1 and S2 [file research.0869.f1.docx]

# Supplementary materials

**We provide additional data and supplementary experiments, including:**

1. Comparison between U-structure and E-structure paradigms (**Appendix S1**).
2. Detailed training strategies (**Appendix S2**).
3. Additional details on computational efficiency and resource utilization (**Appendix S3**).
4. Detailed ablation studies on MLKConv design and hyperparameter sensitivity (**Appendix S4**)
5. Visual comparison of attention maps for U-shaped and E-shaped structure models (**Appendix S5**)
6. Robustness to common imaging artifacts and domain shifts (**Appendix S6**).

**Appendix S1**

Comparison between U-structure and E-structure paradigms

To further illustrate the differences between conventional U-shaped decoder structure and proposed E-shaped aggregation structure, we present a theoretical comparison of two paradigms. As shown in Figure S1(A), a typical U-shaped architecture is shown. A typical U-shaped architecture uses a symmetric encoder–decoder design, where skip connections are employed to fuse encoder features into decoder. The decoding process can be viewed as a recursive chain of stage-wise predictions:

$F_{n}=\text{Φ}_{n}(U(F_{n+1})\odot E_{n})$,

where *F_n_*_+1_ is the output of deeper decoder stage, *U*(⋅) denotes an upsampling operator, *E_n_* ​ is the encoder feature from *n*-th layer, and *Φ_n_*(⋅) represents the non-linear decoding operation at the *n*-th decoder level, and denotes the skip connection. The corresponding feature propagation path is defined as:

$E_{N}\to F_{N}\to F_{N-1}\to\cdots\to F_{1}\to Y_{U}$,

where *Y_U_* denotes the final segmentation map. This chain-like structure deepens the optimization path and may introduce semantic mismatches or feature distortion through skip connections. The entire inference process can be regarded as a Markov chain, where each state relies on the previous one, potentially leading to information attenuation.

In contrast, E-shaped structure, illustrated in Figure S1(B), simplifies the overall process as:

$Y_{E}=\text{Ψ}_{E}\left(\oplus_{n=1}^{N} U_{n}\left(E_{n}\right)\right)$,

where *E_n_* denotes the output from the *n*-th encoder stage, *U_n_* is the corresponding upsampling operation (e.g., UpConv), ⊕ denotes the multi-scale feature aggregation operation (e.g., point-wise addition), and $\text{Ψ}_{E}(\cdot)$​ is a refinement function (e.g., MLKConv) applied to the aggregated features. This formulation introduces parallel information flows:

$\{E_{1},E_{2},\cdots,E_{n}\}\to Y_{E}$,

where *Y_E_* denotes the final segmentation map. Bypassing the deep dependency chain of conventional decoders, each encoder feature directly contributes to the final prediction, reducing the optimization depth and improving both gradient flow and feature preservation, while also enabling multi-scale redundancy. This redundancy allows features from diverse receptive fields to collaboratively enhance fine-grained anatomical structures. Furthermore, unlike the composite, non-convex optimization posed by multiple decoder stages, this formulation results in a flatter optimization landscape. This is especially beneficial for medical segmentation tasks, where training samples are limited and overfitting is a concern. The parallel aggregation paradigm also eliminates cumulative interpolation artifacts and facilitates more robust boundary and context modeling, which we further reinforce with the proposed MLKConv refinement module.


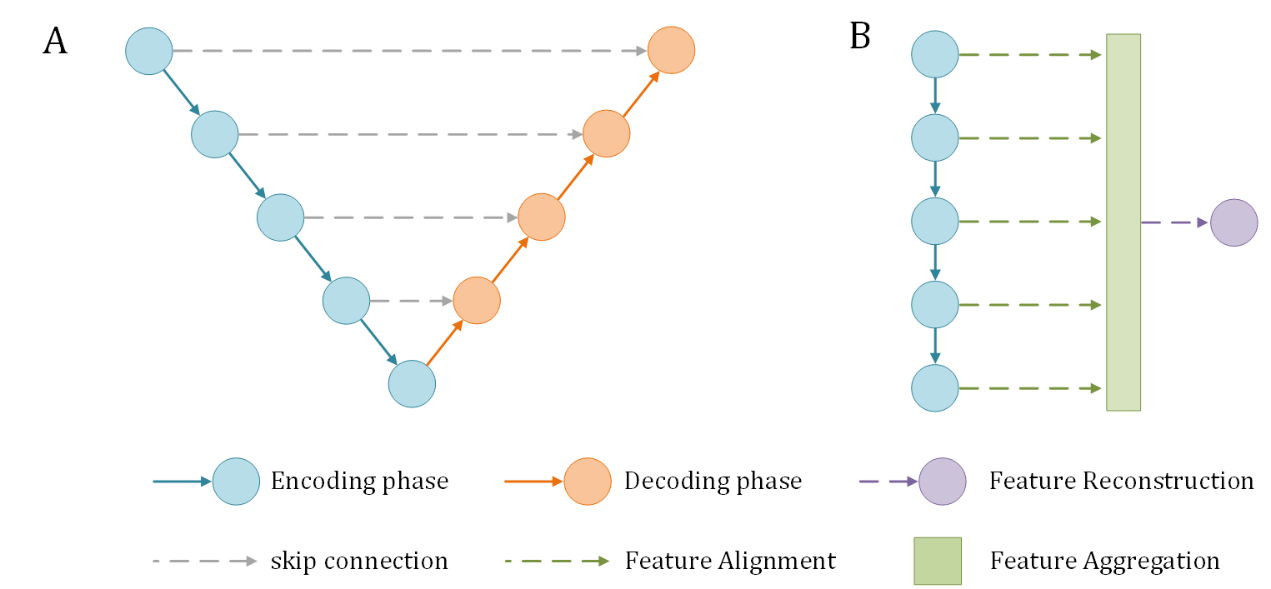


**Figure S1.** Comparison of U-shaped and E-shaped segmentation structures. (A) Traditional U-shaped architecture with symmetric encoder-decoder paths and skip connections. Feature refinement occurs progressively through a chain of decoding stages. (B) Proposed E-shaped architecture. Features from all encoding stages are upsampled and aligned to the original resolution, followed by a unified feature aggregation process. A dedicated feature reconstruction module (e.g., MLKConv) is applied to refine the aggregated features. This design supports parallel information flow and efficient multi-scale integration.

**Appendix S2**

**Detailed training strategies**

This part provides detailed training configurations, as well as the underlying rationale for the different loss weightings applied to 2D and 3D models.

For 2D E-SegNet training, Synapse dataset uses random flipping, affine transformation, and color jittering. ACDC dataset uses random flipping and rotation. Kvasir-SEG dataset uses random flipping, affine transformation, elastic deformation, and color jittering. BUSI dataset uses random flipping (horizontal and vertical), rotation, brightness and contrast adjustment, as well as CLAHE or gamma correction. Different augmentation strategies are selected for each dataset based on imaging modality and data scale to enhance model generalization. A fixed random seed is used for all augmentations to ensure reproducibility and consistency in optimization. For 3D E-SegNet training and preprocessing, we strictly follow the configuration and pipeline provided in nnFormer, which is the standard implementation commonly used in current 3D medical image segmentation models.

Table S2 presents the training configurations for each dataset. Considering low contrast and variable quality of ultrasound images, BUSI dataset uses a customized loss function *L_bhy_*, which combines binary cross-entropy (*L_ce_*, α = 0.3), Dice loss (*L_dice_*, β = 0.3), and focal loss (γ = 0.4), along with an additional boundary-aware term that emphasizes segmentation precision near lesion edges. The boundary component is constructed by detecting contour regions through morphological operations and applying additional penalty weights to misclassified boundary pixels. This hybrid formulation improves both region-level overlap and boundary localization, which are particularly critical in ultrasound-based tumor segmentation.

For other 2D datasets, a weighted combination of Dice loss (*L_dice_*) and cross-entropy loss (*L_ce_*) is used. The choice of different loss weightings between 2D and 3D variants is motivated by task-specific considerations. In 2D segmentation, increased weight is assigned to Dice loss to address severe class imbalance typically present in individual slices, while cross-entropy is retained to improve boundary-level precision. In contrast, 3D segmentation benefits from inherent spatial continuity and reduced label sparsity, making equal weighting more suitable for balancing global volume consistency and voxel-wise classification accuracy. This is also consistent with the loss function design used in most studies on the same datasets.

**Table S1.** Detailed configurations and hyperparameters for model training.

|  |  | | |  |  | | | | |
| --- | --- | --- | --- | --- | --- | --- | --- | --- | --- |
| Configurations | 2D E-SegNet | | |  | 3D E-SegNet | | | | |
|  |  |  |  |  |  |  |  |  |  |
|  |  |  |  |  |  |  |  |  |  |
|  | Synapse | ACDC | Kvasir-SEG | BUSI | Synapse | | NIH Pancreas | ACDC | Lung |
|  |  |  |  |  |  | |  |  |  |
|  |  |  |  |  |  | |  |  |  |
| Batch size | 8 | 8 | 8 | 8 | 2 | | 1 | 2 | 2 |
| Epoch | 400 | 400 | 500 | 400 | 1000 | | 400 | 1000 | 1000 |
| Learning rate | 1e-4 | 2e-4 | 1e-4 | 1e-4 | 1e-4 | | 1e-4 | 8e-4 | 8e-4 |
| Weight decay | 5e-05 | 1e-4 | 5e-05 | 5e-05 | 3e-5 | | 1e-5 | 3e-5 | 3e-5 |
|  |  |  |  |  |  | |  |  |  |
|  |  |  |  |  |  | |  |  |  |
| Loss | 0.6×*L_dice_* + 0.4×*L_ce_* | | | *L_bhy_* | *L_dice_* + *L_ce_* | | | | |
| Optimizer | AdamW | | | | | | | | |
|  |  | | | | |  | | | |

**Appendix S3**

**Additional details on computational efficiency and resource utilization**

This section provides implementation details and full results regarding computational performance and resource utilization across 2D and 3D segmentation models. All experiments are performed under a consistent hardware configuration (NVIDIA RTX 3090, 24GB) and Python environment. To ensure fair comparison and stable results, the input sizes are standardized (2D: 448×448; 3D: 64×128×128), and a batch size of 1 is used. The average inference speed is calculated based on 500 test images. Table S2 presents the full computational comparison, including parameter count, FLOPs, FPS, memory usage, and GPU utilization.

**Table S2.** Comparison of computational efficiency and resource consumption among different 2D and 3D segmentation models, including parameter size (Param, M), computational complexity (FLOPs, G), inference speed (FPS), memory usage during training and inference (Mem, GB), and GPU utilization (GPU Util, %).

|  | Methods | Param(M) | FLOPs(G) | FPS | Train | |  | Inference | |
| --- | --- | --- | --- | --- | --- | --- | --- | --- | --- |
|  |  |  |  |  | Mem(G) | GPU Util(%) |  | Mem(G) | GPU Util(%) |
| 2D Methods | 2D E-SegNet | 30.88 | 15.77 | 74.63 | 1.83 | 88.69 |  | 1.37 | 72.60 |
|  | AgileFormer | 112.98 | 103.82 | 14.41 | 3.83 | 79.62 |  | 2.88 | 55.10 |
|  | MERIT | 147.9 | 72.83 | 15.74 | 4.09 | 62.67 |  | 2.83 | 43.63 |
|  | MERIT-GCASCADE | 143.35 | 61.72 | 15.74 | 4.17 | 65.69 |  | 3.04 | 53.54 |
|  | 2D D-LKA Net | 101.64 | 79.6 | 13.66 | 3.24 | 77.60 |  | 2.62 | 58.36 |
|  | EMCAD | 26.77 | 19.49 | 48.53 | 1.32 | 73.55 |  | 1.10 | 54.90 |
|  | PVT-GCASCADE | 29.17 | 22.12 | 52.38 | 1.67 | 89.04 |  | 1.46 | 82.60 |
|  | PVT-CASCADE | 35.28 | 27.30 | 54.55 | 1.30 | 81.44 |  | 1.03 | 61.90 |
|  | FCB Former | 52.96 | 120.39 | 26.68 | 3.51 | 85.47 |  | 3.96 | 96.06 |
|  | [SSFormer-L](https://paperswithcode.com/paper/stepwise-feature-fusion-local-guides-global) | 66.22 | 58.00 | 29.44 | 2.14 | 73.06 |  | 1.54 | 54.40 |
|  | MISSFormer | 42.46 | 33.98 | 50.33 | 1.73 | 90.36 |  | 1.42 | 89.60 |
|  | SwinUnet | 27.17 | 24.56 | 73.71 | 1.21 | 86.52 |  | 0.91 | 69.58 |
|  | TransUnet | 96.07 | 88.91 | 40.44 | 2.79 | 97.39 |  | 1.91 | 99.80 |
|  |  |  |  |  |  |  |  |  |  |
| 3D Methods | 3D E-SegNet | 30.39 | 147.2 | 78.62 | 8.25 | 99.96 |  | 8.05 | 100 |
|  | D-LKA Net | 42.35 | 66.96 | 40.10 | 6.31 | 99.66 |  | 6.03 | 100 |
|  | UNETR++ | 42.96 | 63.94 | 39.11 | 5.12 | 98.84 |  | 4.15 | 98.76 |
|  | nnFormer | 150.5 | 213.4 | 64.55 | 8.18 | 98.88 |  | 6.92 | 100 |
|  | Swin UNETR | 62.83 | 384.2 | 14.57 | 16.87 | 77.45 |  | 7.11 | 68.30 |
|  | UNETR | 92.49 | 75.76 | 88.96 | 3.92 | 98.18 |  | 3.03 | 99.28 |

**Appendix S4**

**Detailed ablation studies on MLKConv design and hyperparameter sensitivity**

As shown in Table S3, a component-wise ablation study is conducted on MLKConv module. The results demonstrate that multi-scale kernel sizes and hierarchical dilation significantly contribute to segmentation performance. The combination of GConv kernel sizes [3,5,7,11] and DConv dilation rates [3,5,7,11] achieves the best average DSC in both 2D and 3D E-SegNet. Importantly, adding a ReLU activation to the final GConv layer leads to a notable performance drop, with the average DSC decreasing by 16.33% for 2D E-SegNet and by 3.45% for 3D E-SegNet.

**Table S3.** Component-wise ablation study of MLKConv module for E-SegNet on Synapse dataset. “Kernel size” refers to group convolution kernel size combination used in GConv modules of the first stage in MLKConv, where each branch applies a different kernel. “Dilation rate” denotes the dilation rate combination used in DConv modules of the second stage. “ReLU” indicates whether a ReLU activation is applied after the final GConv layer.

| Kernel size | Dilation rate | ReLU | 2D E-SegNet | |  | 3D E-SegNet | |
| --- | --- | --- | --- | --- | --- | --- | --- |
|  |  |  | DSC(%)↑ | HD95↓ |  | DSC(%)↑ | HD95↓ |
| [3,5,7,11] | [3,5,7,11] | **×** | **86.15** | 14.60 |  | **87.76** | **6.32** |
| [3,3,3,3] | [3,5,7,11] | **×** | 85.13 | 15.32 |  | 86.15 | 7.63 |
| [3,5,7,9] | [3,5,7,11] | **×** | 85.96 | 13.63 |  | 86.65 | 7.24 |
| [3,5,7,11] | [3,3,3,3] | **×** | 85.32 | **11.37** |  | 86.43 | 8.65 |
| [3,5,7,11] | [3,5,7,9] | **×** | 86.03 | 13.23 |  | 87.52 | 6.83 |
| [3,5,7,11] | [3,5,7,11] | √ | 69.82 | 30.15 |  | 84.31 | 11.74 |

As shown in Table S4, a sensitivity analysis is performed on three training hyperparameters: epoch number, learning rate, and batch size. The results show that reducing the epoch number or batch size leads to performance degradation in both 2D and 3D settings. Learning rate variations exhibit inconsistent effects. A lower learning rate may slightly improve HD95, but DSC still tends to decrease, while a higher learning rate generally leads to marginal degradation in both metrics.

**Table S4.** Sensitivity analysis of training hyperparameters for E-SegNet on Synapse dataset.

| Hyperparameter | Choice | 2D E-SegNet | |  | Choice | 3D E-SegNet | |
| --- | --- | --- | --- | --- | --- | --- | --- |
|  |  | DSC(%)↑ | HD95↓ |  |  | DSC(%)↑ | HD95↓ |
| Epoch | 300 | 85.95 | 14.86 |  | 500 | 86.31 | 7.49 |
|  | 200 | 84.33 | 15.60 |  | 250 | 84.26 | 9.56 |
| Learning rate | 1e-3 | 85.53 | 15.32 |  | 1e-3 | 87.06 | 6.95 |
|  | 1e-5 | 85.26 | 13.63 |  | 1e-5 | 86.59 | 7.48 |
| Batch size | 4 | 85.48 | 15.37 |  | 2 | 87.76 | 6.32 |
|  | 2 | 84.53 | 17.23 |  | 1 | 86.42 | 6.83 |

**Appendix S5**

**Visual comparison of attention maps for U-shaped and E-shaped structure models**

As shown in Figure S2, E-shaped structure exhibits more accurate and semantically aligned attention distributions compared to U-shaped model. Owing to its larger effective receptive field, E-shaped model provides more complete coverage of large organs such as the liver, successfully capturing their full spatial extent. For small and anatomically irregular organs like the gallbladder and pancreas, it yields more precise localization, whereas U-shaped model shows minimal or even absent attention in these regions. Furthermore, misplaced attention is observed in U-shaped model around the left and right kidneys, as well as in the spleen, indicating a lack of spatial discrimination.


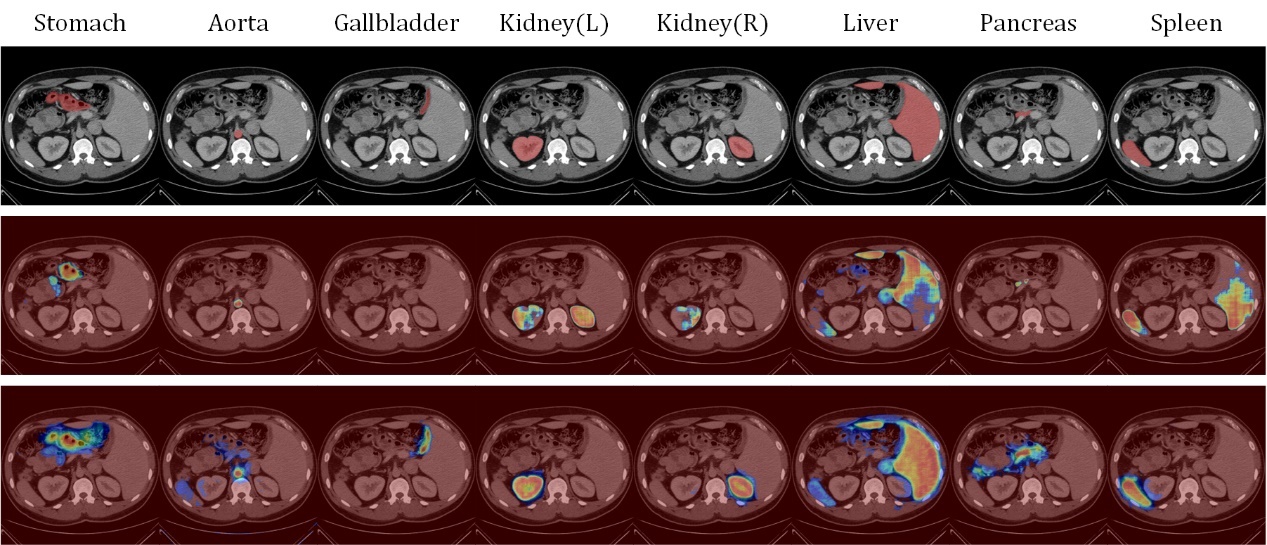


**Figure S2.** Grad-CAM visualization of model attention across eight abdominal organs on Synapse dataset. From top to bottom: (1) Ground truth segmentation masks; (2) attention heatmaps from a traditional U-Net model; (3) attention heatmaps from an E-Net variant that incorporates the proposed E-shaped structure. Both models use the same encoder architecture to ensure a fair comparison. Warmer colors indicate regions with higher model attention.

**Appendix S6**

**Robustness to common imaging artifacts and domain shifts**

To evaluate the adaptability of 2D and 3D E-SegNet in real-world clinical scenarios, we conduct a series of robustness experiments simulating common imaging artifacts and domain shifts. The tested conditions cover typical degradation factors frequently encountered during the acquisition, storage, or transmission of medical images, including Gaussian noise, contrast distortion, and motion blur.

In these experiments, the model parameters remained unchanged from the training phase. During testing, synthetic artifacts of varying intensity are applied to the input images as follows: Gaussian noise with standard deviations σ = 0.05 (mild) and σ = 0.10 (severe); contrast adjustments with gamma values γ = 0.9-1.1 (mild) and γ = 0.8-1.2 (severe); and motion blur with kernel sizes of 3 and 5.

As summarized in Table S3, E-SegNet demonstrates strong robustness under most perturbation conditions. 2D E-SegNet shows minimal degradation in segmentation accuracy under mild contrast variations and motion blur, with a maximum DSC drop of less than 0.6%, indicating good structural adaptability to blurring and brightness fluctuations. However, under severe Gaussian noise (σ = 0.10), our model experiences a substantial decline in performance (approximately 12.3% drop in DSC), revealing its sensitivity to local pixel distribution perturbations.

In comparison, 3D E-SegNet maintains stable performance under both Gaussian noise and motion blur. Nonetheless, it exhibits a pronounced drop in DSC (up to 8.5%) under strong contrast changes (γ = 0.8-1.2). This contrast in behavior can be attributed to differences in receptive field size and spatial context integration between the 2D and 3D architectures. 2D model is more sensitive to localized pixel intensity variations due to its limited receptive field and insufficient contextual fusion, making it more vulnerable to noise. In contrast, 3D model benefits from a larger receptive field and enhanced spatial context integration along the voxel dimension, which helps “smooth out” localized distortions. However, contrast-induced global grayscale shifts can be amplified in models with larger receptive fields, potentially increasing feature extraction errors.

**Table S5.** Performance of 2D and 3D E-SegNet under various image artifacts and domain shifts. All models are evaluated without retraining, using weights obtained from training on clean data. Simulated degradations applied during testing include Gaussian noise (σ = 0.05, 0.10), contrast adjustment (γ = 0.9-1.1, 0.8-1.2), and motion blur (kernel size = 3, 5).

| Artifact Type | Condition | 2D E-SegNet | |  | 3D E-SegNet | |
| --- | --- | --- | --- | --- | --- | --- |
|  |  | DSC(%)↑ | HD95↓ |  | DSC(%)↑ | HD95↓ |
| None (Clean) | - | **86.15** | **14.60** |  | **87.76** | **6.32** |
| Gaussian Noise | σ = 0.05 | 84.03 | 14.67 |  | 87.69 | 6.41 |
|  | σ = 0.10 | 73.85 | 22.03 |  | 87.63 | 8.93 |
| Contrast | γ = 0.9-1.1 | 85.96 | 14.55 |  | 82.46 | 10.46 |
|  | γ = 0.8-1.2 | 85.69 | 14.09 |  | 79.23 | 18.24 |
| Motion Blur | kernel = 3 | 85.98 | 12.28 |  | 85.64 | 7.63 |
|  | kernel = 5 | 85.54 | 15.86 |  | 84.10 | 8.57 |
